# Milder Form of Cobalamin C Disease May Be Missed by Newborn Screening: The Importance of Methylmalonic Acid Assessment

**DOI:** 10.3390/ijns11030077

**Published:** 2025-09-12

**Authors:** Francesca Nardecchia, Agnese De Giorgi, Silvia Santagata, Teresa Giovanniello, Manuela Tolve, Antonio Angeloni, Vincenzo Leuzzi, Francesco Pisani, Claudia Carducci

**Affiliations:** 1 Unit of Child Neurology and Psychiatry, Department of Human Neuroscience, Sapienza University of Rome, Via dei Sabelli 108, 00141 Rome, Italy; agnese.degiorgi@uniroma1.it (A.D.G.); vincenzo.leuzzi@uniroma1.it (V.L.); francesco.pisani@uniroma1.it (F.P.); 2 Unit of Clinical Pathology, Policlinico Umberto I University Hospital, 00161 Rome, Italy; s.santagata@policlinicoumberto1.it (S.S.); t.giovanniello@policlinicoumberto1.it (T.G.); m.tolve@policlinicoumberto1.it (M.T.); 3 Department of Experimental Medicine, Sapienza University of Rome, 00185 Rome, Italy; antonio.angeloni@uniroma1.it (A.A.); claudia.carducci@uniroma1.it (C.C.)

**Keywords:** newborn screening, CblC defect, propionylcarnitine, methylmalonic acid, second-tier test, late-onset

## Abstract

CblC deficiency is the most common intracellular disorder of vitamin B12 metabolism. Expanded newborn screening (NBS) plays a key role in early diagnosis, allowing timely treatment and preventing serious complications. However, traditional first-tier markers—such as propionylcarnitine (C3) and its ratios with other metabolites (e.g., methionine, carnitine, and acetylcarnitine)—have limited sensitivity, particularly for mild forms, leading to missed or delayed diagnoses. In this study, we analyzed data from the NBS Center of the Lazio region (Italy) and identified nine newborns with confirmed CblC deficiency. All were recalled due to abnormalities in C3 or related ratios, along with elevated methylmalonic acid (MMA) levels. Notably, three infants had completely normal C3 levels and ratios during the second screening test, yet they showed MMA levels above the cut-off value (2 µmol/L), enabling a diagnosis of otherwise undetectable mild CblC cases. Our center regularly measures MMA in dried blood spots, even when first-tier markers return to normal on the second sample. This approach allows for early diagnosis and immediate treatment with hydroxocobalamin in patients with mild CblC deficiency, resulting in early intervention, effective metabolic control, and, based on current follow-up, normal neurodevelopmental outcomes. Our findings highlight the essential role of second-tier MMA testing in improving the detection of mild CblC deficiency during NBS.

## 1. Introduction

Methylmalonic acidemia with homocystinuria due to CblC deficiency (OMIM# 277400), the most common intracellular disorder of vitamin B12 metabolism, is an autosomal recessive disease caused by a mutation in the *MMACHC* gene (OMIM* 609831) [1] or by a specific splice-site variant in the neighboring *PRDX1* (OMIM* 176763) associated with an epigenetic form of CblC (epi-CblC) [2]. The MMACHC protein has multiple functions that are not yet fully understood. It can function as a transport protein for cobalamin intermediates and as a catalytic enzyme [3,4,5]. CblC deficiency affects the synthesis of two essential coenzymes: adenosylcobalamin (AdoCbl), a cofactor of mitochondrial methylmalonyl-CoA mutase (EC 5.4.99.2) that converts methylmalonyl-CoA into succinyl-CoA, and methylcobalamin (MeCbl), a cofactor of cytosolic methionine synthase (EC 2.1.1.13) that catalyzes the conversion of homocysteine to methionine (Met) [6]. The biochemical impairment in CblC defects (as well as CblF and CblD) can therefore result in elevated circulating and urinary levels of homocysteine (Hcy) and methylmalonic acid (MMA) [7,8].

The variable onset and spectrum of symptoms in CblC deficiency are well documented. Patients with early-onset disease (usually within the first year of life) may present with feeding difficulties, failure to thrive, hypotonia, seizures, hydrocephalus, microcephaly, developmental delay, cognitive impairment, hematological abnormalities (thrombocytopenia, macrocytic anemia, leukopenia, and neutropenia), renal failure (sometimes hemolytic–uremic syndrome), and ophthalmological involvement [9,10,11]. The late-onset subgroup (usually after four years of age) may present with gait abnormalities, extrapyramidal symptoms, peripheral neuropathy, psychiatric disorders, declining cognitive performance, dementia, leukoencephalopathy, subacute degeneration of the spinal cord, macrocytic anemia, and thromboembolic events [9,11,12,13].

Treatment of CblC deficiency typically includes hydroxocobalamin, betaine, folate/folinic acid supplementation, and carnitine [8].

Expanded newborn screening enables the early diagnosis of CblC deficiency and the initiation of timely treatment, which is essential to prevent serious complications of the disease.

Current guidelines recommend using propionylcarnitine (C3) and the C3/C2 ratio as primary markers for early-onset CblC deficiency screening. However, these markers have low specificity, and their sensitivity for detecting mild or late-onset cases remains uncertain. They may also detect neonatal metabolic disturbances caused by maternal B12 deficiency. Incorporating second-tier tests, such as total Hcy (tHcy) and MMA in dried blood spots (DBSs), enhances specificity and helps distinguish CblC defects from other disorders [8].

Here, we report the diagnosis through newborn screening of three CblC patients, who showed persistent MMA elevation despite the normalization of C3 levels and related ratios.

## 2. Materials and Methods

From December 2010 to January 2020, a total of 308,954 newborns underwent screening at the Lazio regional center. The XEVO-TQMS tandem mass spectrometry (MS/MS) system (Waters Corporation, Milford, MA, USA) in combination with the Neogram derivatized MS/MS kit (PerkinElmer, Waltham, MA, USA), which ensures high sensitivity and specificity in identifying key metabolic markers, was used. For the assessment of propionate metabolism disorders, the established cut-off values were as follows: C3 at 4.38 µmol/L, a C3/C2 ratio at 0.13, a C3/C0 ratio at 0.14, and a C3/Met ratio at 0.22, corresponding to the 98.8%ile of the normal population, except for C3/C0, which was set at the 99.8%ile. The cut-off percentiles were defined by taking into account the distribution of values in the affected population, as suggested by McHugh et al. [14]. Newborns with abnormal levels in any one of these parameters undergo further biochemical analysis to confirm or rule out potential metabolic conditions.

Subsequent evaluations include liquid chromatography–tandem mass spectrometry (LC-MS/MS) to measure MMA and methylcitric acid (MCA), along with high-performance liquid chromatography (HPLC) to quantify tHcy concentrations in the same DBS sample.

**Figure 1:**
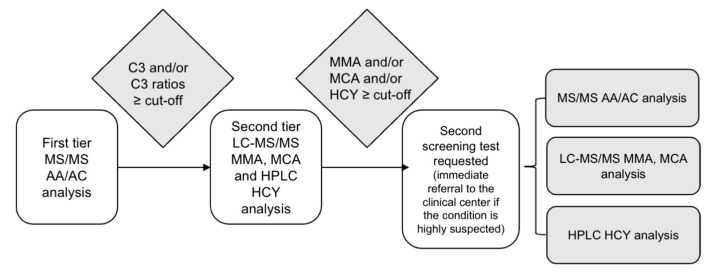
Algorithm used by the Lazio region’s Newborn Screening Center to assess propionate metabolism disorders. Legend = AAs: Amino Acids; AC: Acylcarnitine; HCY: Homocysteine; HPLC: High-Performance Liquid Chromatography; LC: Liquid Chromatography; MS/MS: Tandem Mass Spectrometry; MCA: Methylcitric Acid; MMA: Methylmalonic Acid.

If an elevated MMA level is identified in the initial DBS, a second DBS is requested to confirm the persistence of the abnormality. Importantly, MMA analysis is performed on the second DBS even when C3, C3/C0, C3/C2, and C3/Met levels are within normal ranges, to ensure that mild or late-onset cases are not missed. The algorithm used is shown in Figure 1.

## 3. Results

Out of 308,954 screened newborns, 9 were diagnosed with CblC deficiency (Table 1).

Three of these patients had completely normal C3 levels and C3 ratios at the second screening (performed between 8 and 20 days of life), while their MMA levels were above the cut-off value of 2 µmol/L, ranging from 3.1 to 7.6 µmol/L (Table 2; ID5, ID6, and ID8).

Met levels were normal in all three patients at both the first and second screening tests. Molecular analysis confirmed the diagnosis of CblC deficiency in all cases (Table 3).

The reported cases were classified as a late-onset/mild form of the disease, based on mild biochemical alterations, unremarkable neonatal neurological examination, and consistent genotype [1].

**Figure 2:**
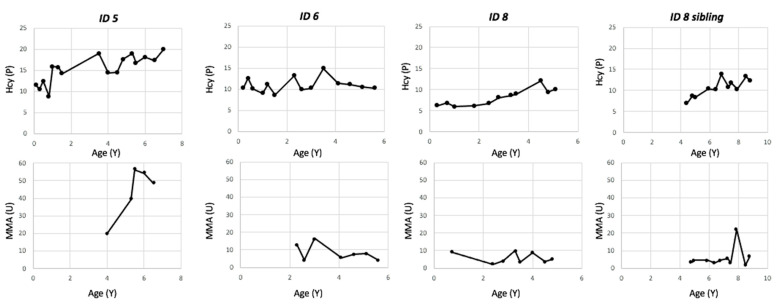
Longitudinal biochemical follow-up of patients with late-onset/mild CblC from treatment onset. Legend = HCY (P): Plasmatic Homocysteine; MMA (U): Urinary Methylmalonic Acid.

Additionally, an older sibling of one CblC patient, who had not undergone expanded NBS, was diagnosed with CblC at the age of 4 years despite having normal C3 levels. Vitamin B12 levels before starting treatment were normal or above normal (pg/mL, ID 5: 1089; ID 6: 681; ID 8: 1293). Patients were treated with hydroxocobalamin monotherapy, keeping plasma tHcy levels below 20 μmol/L and urinary MMA levels below 60 mmol/mol creatinine. Two patients diagnosed through newborn screening (ID 6 and ID 8), along with the sibling of ID 8, responded well to oral hydroxocobalamin administration, maintaining good metabolic control (tHcy < 15 µmol/L and urinary MMA < 23 mmol/mol creatinine) (Figure 2) and improving treatment compliance. All patients show normal developmental outcomes (Table 4), except for the sibling of ID8, who shows a mild specific learning disorder. Ophthalmological evaluation of ID6, at the age of 4, detected bilateral temporal lens subluxation. Exome sequencing is ongoing for this patient since ectopia lentis has not yet been reported in CblC [15].

## 4. Discussion

Propionylcarnitine (C3) is the primary marker for various propionate metabolism disorders, such as methylmalonic acidemia, propionic acidemia, congenital cobalamin defects, and secondary vitamin B12 deficiency due to maternal vitamin B12 deficiency [16].

Since the introduction of expanded newborn screening using tandem mass spectrometry, determining the optimal C3 cut-off values for detecting propionate metabolism disorders has proven challenging. High cut-off values increase the risk of false negatives, while low cut-off values lead to more false positives and a higher recall rate. Additionally, some cobalamin metabolism disorders may not result in significant C3 elevations, limiting detection when C3 is used alone. To enhance diagnostic accuracy and reduce false positives, many laboratories complement C3 measurements with acylcarnitine ratios [14,16,17,18]. Methionine (Met) levels are routinely measured in NBS. In cobalamin C defects, Met levels are typically low or low–normal due to impaired methylcobalamin (MeCbl) production. However, low Met alone is not a specific marker for these conditions, as it can also be observed in healthy neonates (due to low protein intake after birth) and in individuals with methylenetetrahydrofolate reductase (MTHFR) deficiency. Nevertheless, one study by Weisfeld-Adams et al. [16] showed that low Met levels could serve as an additional secondary analyte, improving specificity and enhancing positive predictive value. Finally, Malvagia et al. [19], building on studies that identified elevated 3-hydroxypalmitoleoyl-carnitine (C16:1-OH) in the acylcarnitine profile of patients with propionic acidemia or methylmalonic acidemia, identified heptadecanoylcarnitine (C17) as a novel biomarker for these conditions, potentially enhancing the accuracy of NBS, particularly in laboratories where second-tier tests are not implemented.

Due to the broad range of clinical manifestations or biological variation in the CblC defect, some cases may go undetected during NBS.

Wilcken et al. [20], in their evaluation of NBS efficacy using tandem mass spectrometry, reported a case of cobalamin C deficiency with negative NBS results. At five weeks of age, the infant presented with hypotonia and failure to thrive. A retrospective analysis revealed an increased C3/C2 ratio (0.3; cut-off 0.25). This ratio was subsequently adopted as a primary diagnostic variable. In subsequent years, the same authors [21] identified 11 missed cases with analyte values below action limits, reflecting milder phenotypes or biological variations in the disorders. These cases, including another instance of CblC deficiency, did not lead to further protocol changes as the risk of increasing the false-positive rate to unacceptable levels was considered too high.

Similarly, an analysis of the eight-year newborn screening experience in North Carolina (1997–2005), involving 944,078 newborns, identified 1 patient with CblC deficiency who died at 22 days of life. In this case, the expanded NBS results (C3 and the C3/C2 ratio) were classified as borderline according to the diagnostic cut-off used at the time, which delayed reporting of the results. Subsequently, the diagnostic cut-offs were lowered to improve detection efficiency [22].

La Marca et al. [23] reported on the NBS experience for over 40 inborn errors of metabolism in the Tuscany region (Italy) and highlighted advancements in reducing false positives and negatives. For C3, the initial cut-off of 3.3 µmol/L (+2 SD) established during the pilot study was later raised to 5.65 µmol/L (+4.5 SD) to reduce recall rates. Despite this adjustment, between 2004 and 2006, C3 recalls still constituted 22% (124 of 564) of total recalls. To address this issue, a second-tier test was introduced, measuring MMA and 3-hydroxypropionic acid levels on neonatal DBS, which improved the positive predictive value of C3 from 4% to 100%, allowing the cut-off to be lowered back to 3.3 µmol/L [23].

The same paper documented a case of CblC defects that was not detected by NBS (C3: 4.20 µmol/L; cut-off: 5.65 µmol/L). A retrospective analysis of the DBS revealed elevated levels of 3-hydroxypropionic acid and MMA. Had the cut-off of 3.3 µmol/L been applied, the patient would have been diagnosed through the NBS process.

Ahrens-Nicklas et al. [24] reported the case of a male infant who presented with low carnitine (C0) levels on newborn screening. Subsequent metabolic testing confirmed persistently low carnitine and elevated C3, Hcy, and MMA, which is consistent with a genetically confirmed cobalamin defect. Treatment with hydroxocobalamin and levocarnitine led to a significant improvement in his metabolic profile. By 30 months of age, the patient maintained good metabolic control, exhibiting only pseudostrabismus, mild myopic astigmatism, and a speech delay. In the state where the NBS was conducted, the C3/C2 ratio is reported only if C3 levels are elevated, which was not the case for this patient. However, retrospective analysis revealed that the C3/C2 ratio was indeed elevated, suggesting that this ratio could serve as a valuable marker, even when C3 levels appear normal.

To increase positive predictive value, our NBS program uses C3/C2, C3/C0, and C3/Met ratios and measures MMA, MCA, and tHcy in DBS as second-tier tests. The introduction of second-tier testing for MMA and tHcy in the Italian NBS program has enabled a reduction in the C3 cut-off (median 4.7 µmol/L) without increasing unnecessary recalls, thus minimizing false negatives, especially in late-onset CblC cases. This has significantly improved the sensitivity of the screening, allowing for earlier diagnoses [25].

Our center’s experience highlights that a subset of patients with CblC deficiency—particularly those with milder or late-onset forms—may be missed if newborn screening relies solely on C3 levels and related ratios. The systematic inclusion of MMA measurement on dried blood spots (DBSs), even in newborns whose primary markers normalized on re-testing, enabled the identification of three affected infants, as well as a previously undiagnosed older sibling, with mild phenotypes. These findings underscore the importance of extended screening strategies to improve detection sensitivity without increasing false-positive rates. The implementation of second-tier testing for MMA and tHcy at our center has resulted in a significant reduction in the false-positive rate for CblC/D to 0.084%, along with an improvement in the positive predictive value (PPV) to 8.4%, which further increases to 57% when including cases associated with acquired vitamin B12 deficiency.

However, the long-term prognosis of patients with attenuated phenotypes detected through NBS remains uncertain. Published studies indicate that clinical manifestations in mild forms often emerge after 4 years of age, with a mean age of onset around 15 (±9.2) years [26]. Thus, preserved developmental outcomes in early childhood do not preclude the possibility of later complications. This uncertainty justifies both the early initiation of treatment and ongoing clinical and biochemical monitoring, even in cases with initially mild or nonspecific biochemical profiles.

The choice of oral hydroxocobalamin in some patients was guided by considerations of feasibility and treatment adherence. Importantly, all patients treated orally maintained homocysteine levels below 15 μmol/L and urinary methylmalonic acid (MMA) within the normal range. This underscores the need to tailor treatment intensity to disease severity and biochemical profile, while recognizing that parenteral hydroxocobalamin remains the more effective therapeutic option.

Finally, our findings underline the broader challenges of newborn screening for late-onset metabolic disorders. While early detection provides opportunities to prevent or delay disease manifestations, it also raises complex questions about the optimal timing and intensity of treatment, especially in individuals who may remain asymptomatic for years. Future studies with longer follow-up and larger cohorts will be essential to clarify the natural history of attenuated forms, define treatment thresholds, and establish evidence-based guidelines for long-term management.

## Figures and Tables

**Table 1:** Newborn screening results of patients diagnosed with cobalamin C deficiency.

ID	Age (h)	C3 (μmol/L)	C3/Met	C3/C2	C3/C0	Met (μmol/L)	C16:1OH	MMA (μmol/L)	MCA (μmol/L)	tHcy (μmol/L)
**cut-off 2–15 days**		4.38	0.219	0.18	0.14	<10.5	0.18	2	1	3.7
1	48	**8.5**	**1.25**	**0.43**	**0.24**	**7.0**	**0.34**			
2	72	**6.4**	**0.45**	0.13	0.09	14.1	0.03	**8**		
3	72	**10.3**	**1.08**	**0.22**	**0.15**	**9.6**	0.13	**38**		**25**
4	72	**8.6**	**1.5**	**0.28**	**0.27**	**5.7**	**0.18**	**49**		**31**
5	72	4.3	**0.23**	0.14	**0.2**	18.7	0.06	**24.6**	<LOQ	**7.4**
6	72	**4.9**	**0.22**	0.14	**0.14**	21.9	0.06	**5.81**	<LOQ	**4.3**
7	48	**9.0**	**1.19**	**0.27**	**0.25**	**5.6**	**0.19**	**59**	<LOQ	**49.4**
8	48	3.9	0.14	0.11	**0.14**	27.5	0.04	**14.4**	0.72	**7.14**
9	72	**11.8**	**1.93**	**0.32**	**0.26**	**6.1**	**0.18**	**80**	**2**	**68.2**

Legend = LOQ: limit of quantification. Bold values ≥ cut-off.

**Table 2:** Second screening test in three patients with normal C3 levels and C3 ratios and elevated MMA levels.

ID	Age (h)	Age (Days)	C3 (μmol/L)	C3/Met	C3/C2	C3/C0	Met (μmol/L)	C16:1OH	MMA (μmol/L)
**cut-off 2–15 days**			4.38	0.219	0.18	0.14	<10.5	0.18	2
**cut-off >15 days**			3.13	0.118	0.14	0.057	<11.5		2
5	240	10	1.8	0.09	0.08	0.08	20.4	0.03	**8.01**
6	480	20	1.4	0.05	0.07	0.04	30.2	0.02	**3.11**
8	192	8	1.7	0.09	0.08	0.07	18.9	0.02	**7.6**

Legend = bold values ≥ cut-off.

**Table 3:** Results of molecular analysis of three patients with late-onset/mild form of CblC deficiency.

ID		GRCh37/hg19 HGVS	Clinvar ID	RS ID	ACMG Class	Ref	Genotype
5	ALLELE 1	NM_015506.3 (MMACHC): c.271dup NP_056321.2:p.Arg91fs	1421	rs398124292	V (Pathogenetic)	PMID: 33562640, 36338977	NM_015506.3 (MMACHC): [271dup];[347T>C]
	ALLELE 2	NM_015506.3 (MMACHC): c.347T>C NP_056321.2:p.Leu116Pro	1422	rs121918240	IV (Likely Pathogenetic)	PMID: 20301503, 20924684	
6	ALLELE 1	NM_015506.3 (MMACHC): c.440G>C NP_056321.2:p.Gly147Ala	203828	rs140522266	V (Pathogenetic)	PMID: 20301503, 25398587	NM_015506.3 (MMACHC): [347T>C];[440G>C]
	ALLELE 2	NM_015506.3 (MMACHC): c.347T>C NP_056321.2:p.Leu116Pro	1422	rs121918240	IV (Likely Pathogenetic)	PMID: 20301503, 20924684	
8	ALLELE 1	NM_015506.3 (MMACHC): c.271dup NP_056321.2:p.Arg91fs	1421	rs398124292	V (Pathogenetic)	PMID: 33562640, 36338977	NM_015506.3 (MMACHC): [271dup];[440G>C]
	ALLELE 2	NM_015506.3 (MMACHC): c.440G>C NP_056321.2:p.Gly147Ala	203828	rs140522266	V (Pathogenetic)	PMID: 20301503, 25398587	

This table presents the molecular findings of three patients diagnosed with the mild form of CblC deficiency. The allelic variants of the MMACHC gene were identified, including pathogenic and likely pathogenic mutations, along with associated HGVS notations, ClinVar IDs, and ACMG classification. The patients’ genotypes, reference sequences, and relevant literature references (PMID) are also provided.

**Table 4:** Clinical outcome and treatment of patients with late-onset/mild CblC.

ID	Current Age (y)	IQ	Developmental Outcome and/or Academic Performance	Ophthalmological Assessments	Cardiological Assessments	Renal Function Assessment	Hydroxocobalamin Treatment (µg)	Treatment Frequency
5	7.2	NA	N	N	N	N	10,000 (im) *	weekly
6	7.3	101	N	EL ^#^	N	N	3000 (po)	daily
8	5.6	100	N	N	N	N	2000 (po)	daily
ID8 sibling	9.6	93	Mild SLD	N	N	N	5000 (po)	daily

Legend = EL: ectopia lentis; N: normal; NA: not available; im: intramuscular; po: by mouth; SLD: specific learning disorder. * dosage increased at the last follow-up to 20,000 µg for poor metabolic control. ^#^ exome sequencing ongoing to exclude other genetic causes of EL.

## Data Availability

The original contributions presented in this study are included in the article.

## Abbreviations

The following abbreviations are used in this manuscript:

AAs	Amino Acids
AC	Acylcarnitine
AdoCbl	Adenosylcobalamin
C0	Carnitine
C2	Acetylcarnitine
C3	Propionylcarnitine
C16:1OH	3-Hydroxypalmitoleoyl-carnitine
C17	Heptadecanoylcarnitine
CblC	Cobalamin C Defect
DBS(s)	Dried Blood Spot(s)
Hcy	Homocysteine
HPLC	High-Performance Liquid Chromatography
LC	Liquid Chromatography
MCA	Methylcitric Acid
MeCbl	Methylcobalamin
Met	Methionine
MMA	Methylmalonic Acid
MS/MS	Tandem Mass Spectrometry
NBS	Newborn Screening
tHcy	Total Homocysteine
